# Supplementary material for: Active control of flexible spacecraft in orbit based on partial differential equations
Source: PLoS One. 2025 Sep 10;20(9):e0331610. doi: 10.1371/journal.pone.0331610 (PMC12422462; doi:10.1371/journal.pone.0331610)
Supplement: S1 Text — (PDF) [file pone.0331610.s001.pdf]

```
clear

clc;

close all;

clear all;


  %********************

  % Flexible satellite withou control

  %************************************

nx=50; % length of space domain

nt=8*10^4; % length of time domain


tmax=50; % time of simulation

  L=10;

  Ttr=80; % sampling for drawing

  Ttr1 =1*10^3; % sampling for drawing


  dx=L/(nx -1); % spacing step

  dt=tmax/(nt -1); % time step


% create matrix to save data contains

  w = zeros (nx ,nt);v=w;

  u = zeros (nt ,1);

  w_3D= zeros (Ttr ,nx);v_3D=w_3D;

  t_3D= zeros (Ttr ,2*nx -1);

  revise_free= zeros (Ttr ,nx -1);


  % parameters
```

```
  m=20;
  A=0.12;
% E=5*10^8;
E=6.894*10^10;
% rho=5.24;
rho=2.7*10^3;
I=1.734*10^-7;
  gamma1=0.005;
rhoA=rho*A;
  EI=E*I;

  % initial condition
for i=1:nx
  w(i,1)=( nx -i )*dx *0.3;
end

  w(:,2)=w(:,1);
w(:,3)=w(:,1);

  for i=1:nx
  v(i,1)=( i-1)*dx*0.3;
  end
  v(:,2)=v(:,1);
  v(:,3)=v(:,1);

  wl_free= zeros (nt ,1);vl_free=wl_free;
  wl_free(1)=w(1,1);wl_free(2)=wl_free(1);
```

```
  vl_free(1)=v(nx ,1);vl_free(2)=v(1);
  w_L_free= zeros (nt ,1);
  w_L_free(1)=w(nx ,1);
  w_L_free(2)=w(nx ,1);

%main cycle

for j=3:nt -1

  for i=3:nx -2

  wxxxx=( w(i+2,j) -4*w(i+1,j)+6*w(i,j) -4*w(i-1,j)+w(i-2,j) )/dx^4;
  dw=( w(i,j)-w(i,j-1) )/dt;
  vxxxx=( v(i+2,j) -4*v(i+1,j)+6*v(i,j) -4*v(i-1,j)+v(i-2,j) )/dx^4;
  dv=( v(i,j)-v(i,j-1) )/dt;

  w(i,j+1)=2*w(i,j)-w(i,j-1)+(  -EI*wxxxx - gamma1*dw )*dt^2/rhoA;
  v(i,j+1)=2*v(i,j)-v(i,j-1)+( -EI*vxxxx - gamma1*dv )*dt^2/rhoA;
  end

  w(1,j+1)=3*w(3,j+1) -2*w(4,j+1);
  w(2,j+1)=2*w(3,j+1)-w(4,j+1);
v(nx ,j+1) =3*v(nx -2,j+1) -2*v(nx -3,j+1);
  v(nx -1,j+1) =2*v(nx -2,j+1)-v(nx -3,j+1);
  wxxxl =( w(nx ,j) -3*w(nx -1,j)+3*w(nx -2,j)-w(nx -3,j))/dx^3;
  vxxxl=( v(4,j) -3*v(3,j)+3*v(2,j)-v(1,j) )/dx^3;
  S(j)=wxxxl - vxxxl;
```

```
  Q(j)= wxxxl;
  R(j)= vxxxl;
  v(1,j+1)=w(nx ,j+1);
w(nx -1,j+1)=w(nx ,j+1);
v(2,j+1)=v(1,j+1);

wl_free(j)=w(1,j+1);
vl_free(j)=v(nx ,j+1);
  w_L_free(j)=w(nx ,j+1); % w(L/2,t)

% saving data for drawing
  if mod(j-1,nt/Ttr)==0;
end
  end
  w_3D (1,:)=w(:,1)';
  v_3D (1,:)=v(:,1)';
  for i=1:nx
  t_3D(:,i)=w_3D(:,i);
  t_3D(:,nx+i)= v_3D(:,i);
  end
% to reduce the nodes of original nx
  for i=1:2*nx -1
  if mod(i,2)==0
  revise_free(:,i/2)= t_3D(:,i);
  end
  end
```

```
% make a draw

  figure (1);
  surf ( linspace (-L,L,nx -1),t_tr , revise_free); view ([60 35]);
title ('无控制下柔性软管的位移') ;
ylabel ('t [s]');
xlabel ('x[m]') ;
zlabel ('w(x,t) [m]') ;


figure (2);
surf ( linspace (-L,L,nx -1),t_tr , revise_control); view ([60 35]);
  title ('控制下柔性软管的位移') ;
ylabel ('t [s]') ; xlabel ('x [m]') ; zlabel ('w(x,t) [m]') ;
%
figure (3);
    subplot (211);
  plot ( linspace (0,tmax ,nt),wl_free);
xlabel ('t [s]') ; ylabel ('w(l,t) [m]') ;
subplot (212);
plot ( linspace (0,tmax ,nt),wl_control);
  xlabel ('t [s]') ; ylabel ('w(l,t) [m]') ;
%
  figure (4);
  subplot (211);
  plot ( linspace (0,tmax ,nt),w_L_free);
  xlabel ('t [s]') ; ylabel ('w(l/2,t [m]') ;
```

```
subplot (212);

plot ( linspace (0,tmax ,nt),w_L_control);

  xlabel ('t [s]') ; ylabel ('w(l/2,t) [m]') ;

%

figure (5);

  subplot (211);

  plot ( linspace (0,tmax ,nt),vl_free);

xlabel ('t [s]') ; ylabel ('w(0,t) [m]') ;

subplot (212); plot ( linspace (0,tmax ,nt),vl_control);

xlabel ('t [s]') ; ylabel ('w(0,t) [m]') ;
```
