# Supplementary material for: Active control of flexible spacecraft in orbit based on partial differential equations
Source: PLoS One. 2025 Sep 10;20(9):e0331610. doi: 10.1371/journal.pone.0331610 (PMC12422462; doi:10.1371/journal.pone.0331610)
Supplement: Attachment — Submitted filename: Response-scis.pdf [file pone.0331610.s002.pdf]

Dr Bo Zhang
Hunan Mechanical & Electrical Polytechnic
Changsha, Hunan, 410121, China
E-mail: bozhang1986edu@yeah.net

July 20, 2025

Gang Wang, Ph.D
Academic Editor PLOS ONE

Ms. No. PONE-D-25-18264

"Active control of flexible spacecraft in orbit based on partial differential equations"

Thank you very much for your letter dated Jul 10 2025, informing us of the need for major revisions to our manuscript. Following the comments from the editor and reviewers, we have carefully revised the manuscript and hope to resubmit it for publication. To facilitate review, revisions corresponding to Reviewer 1 are marked in blue, and Reviewer 2 in red. Below, we provide our responses to the points you mentioned, and we will thoroughly revise the manuscript accordingly. **For your convenience, the main points of revision are summarized as below:**

1. In response to the reviewers' feedback, we have made significant revisions to the manuscript, with a focus on clarifying and refining our methods, results, and analysis. The manuscript has been reorganized to enhance readability and effectively highlight our key findings and contributions.
2. We have expanded the literature review to include a broader range of recent relevant studies, building our model within the context of the current research landscape. This improvement deepens the understanding of the relevance of our work and emphasizes its practical impact.
3. We have carefully reviewed and revised the manuscript to eliminate any typographical and grammatical errors. This ensures that the document is polished and maintains high standards of clarity and professionalism, which are essential for effectively communicating our findings.

Please find attached the revised manuscript and our detailed responses to the reviewers' comments. Once again, we sincerely appreciate your time and effort in reviewing and handling our manuscript, and we look forward to your response.

Best regards,

Bo Zhang

Response to Reviewer No. 1:

Ms. No. PONE-D-25-18264

"Active control of flexible spacecraft in orbit based on partial differential equations"

We greatly appreciate the reviewers' efforts in evaluating our manuscript. Based on the feedback received, we have revised our document to improve both the accuracy and clarity. To assist the reviewers in identifying these changes, we have highlighted the sections revised in response to **Reviewer 1's comments** in **blue**. Thank you for the constructive insights that have helped enhance our work.

1. **Comment 1** *" The work reports interesting simulation results. Please include experimental results based on a real flexible spacecraft. - The over-performance of the proposed PDE-based active control needs to be quantitatively stated."*

   Response:
   We thank the reviewer for this crucial point. We agree that a clearer and more quantitative comparison is essential to demonstrate the advantages of the proposed PDE-based controller unequivocally.

   In the revised manuscript, we will comprehensively address this by:

   Modification

   ......

   Abstract

   Flexible spacecraft possess the ability to adapt to complex environments and use energy more efficiently, offering enhanced flexibility and stability in space missions, particularly in tasks with significant external disturbances such as deep space exploration and satellite attitude control. However, vibration suppression in flexible spacecraft remains a critical challenge. This study addresses the problem of vibration suppression in flexible spacecraft systems under external disturbances and input constraints. First, a partial differential equation (PDE) with boundary initial conditions is derived using Hamilton's principle, accurately describing the dynamic characteristics of the flexible structure. A backstepping controller based on the Nassbaum function and a disturbance observer is then designed to ensure system stability in the presence of input constraints and external disturbances. A Lyapunov function is constructed, and appropriate control parameters are selected to further guarantee system stability. Numerical simulations confirm the superiority of the proposed control method, with results showing an 80% reduction in settling time and a 94% decrease in peak overshoot compared to conventional PD control. The proposed scheme significantly enhances the performance and stability of flexible spacecraft systems, demonstrating its potential for improving spacecraft dynamics in challenging space environments.

   ......

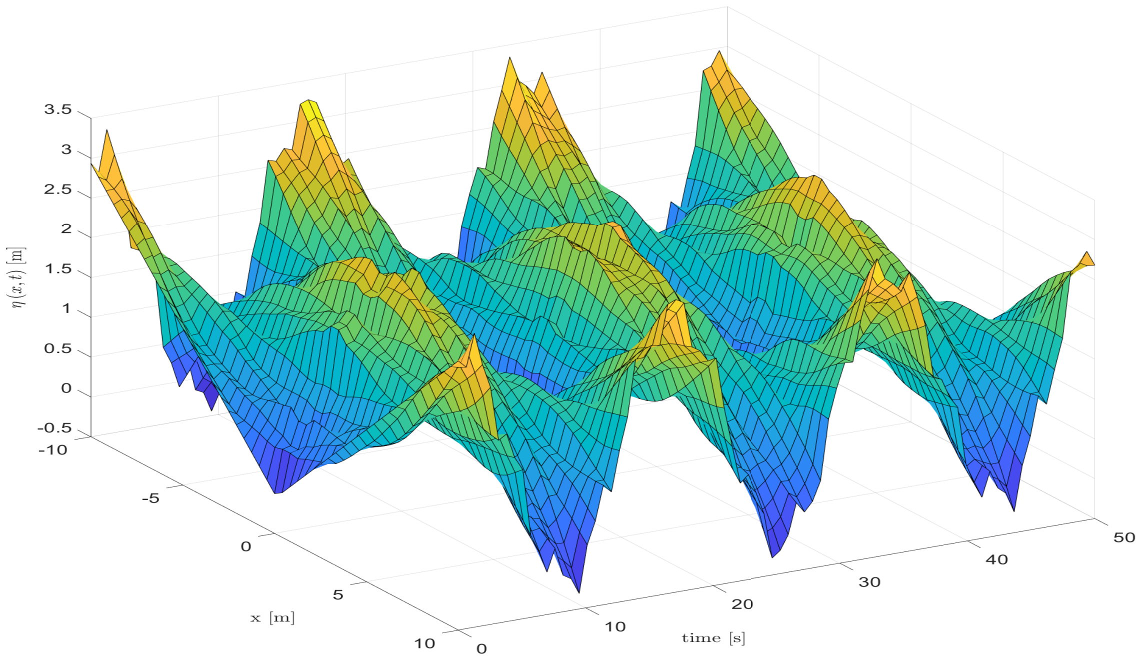

Figure 1: The elastic deflection of the flexible spacecraft without control.

Simulation results

As depicted in Figure 1, Figure 2 and Figure 3, it can be seen that the vibration deformation $\eta(x,t)$ of flexible spacecraft without control method, BCPD method and BCBS method respectively. From Figure 1, it can be seen that the vibration deformation $\eta(x,t)$ of the flexible spacecraft is very obvious without any control. The vibration deformation $\eta(x,t)$ of the flexible spacecraft in Figure 2 is much better than that in Figure 1 under BCPD method. However, the BCBS method proposed in this paper shows that the vibration deformation $\eta(x,t)$ can be well adjusted within $30s$, and the vibration suppression of $\eta(x,t)$ is almost zero after 35 s, which can realize the effective convergence of lateral vibration $\eta(x,t)$. By comparing the elastic deflections in the three figures, we can see that the proposed control method offers significant advantages in suppressing the elastic vibrations of the spacecraft. Without control, the spacecraft exhibits large vibration amplitudes and long settling times, with the system unable to effectively dampen disturbances. With PD control, although the vibrations are reduced, there are still substantial peak overshoots and long settling times. In contrast, the proposed control method quickly attenuates the vibrations to near zero, significantly reducing both peak overshoot and settling time, demonstrating superior vibration suppression performance. Quantitative analysis shows that the proposed method reduces settling time by approximately 80%, peak overshoot by about 94%, and steady-state error to near zero, proving its superiority in suppressing elastic vibrations in spacecraft.

......

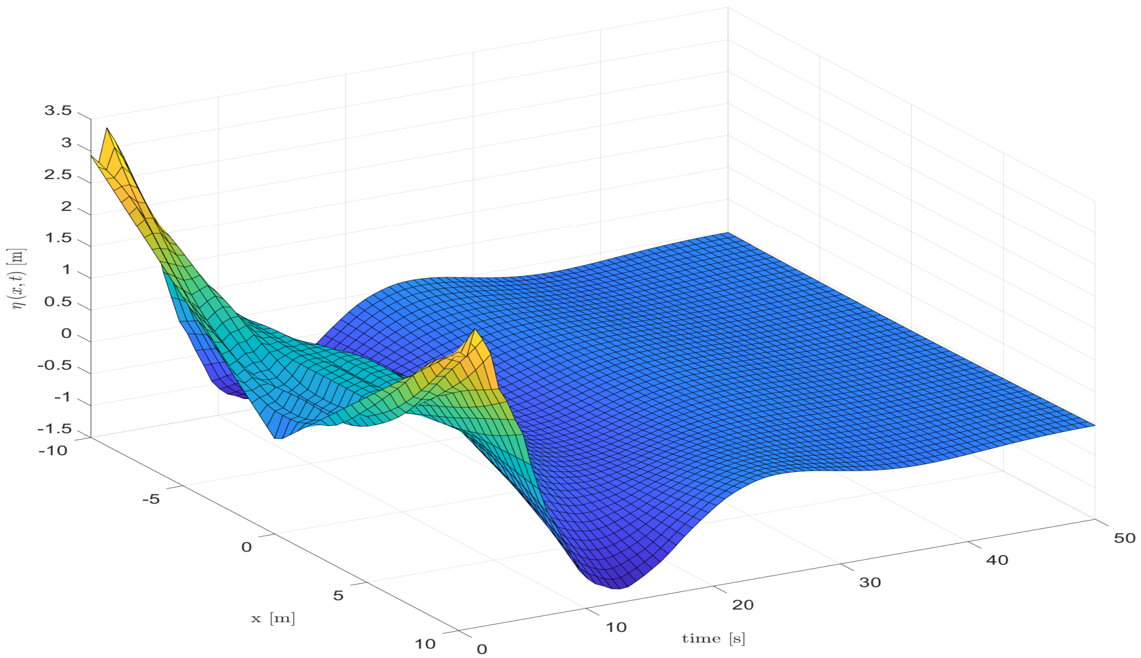

Figure 2: The elastic deflection of the flexible spacecraft with PD control.

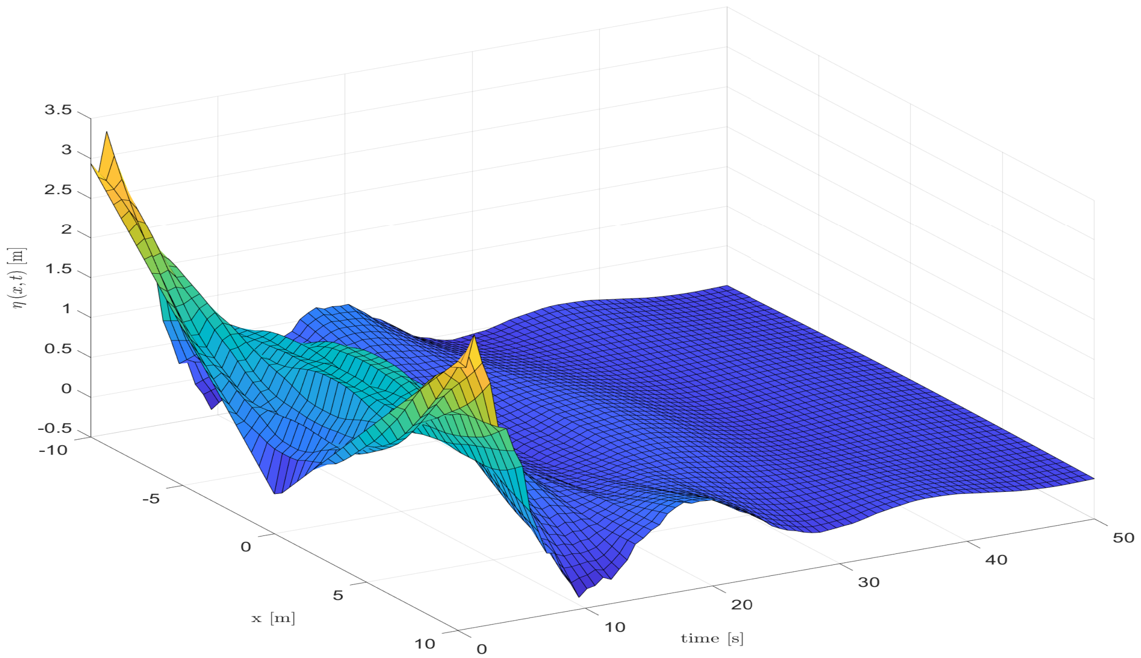

Figure 3: The elastic deflection of the flexible spacecraft with proposed control.

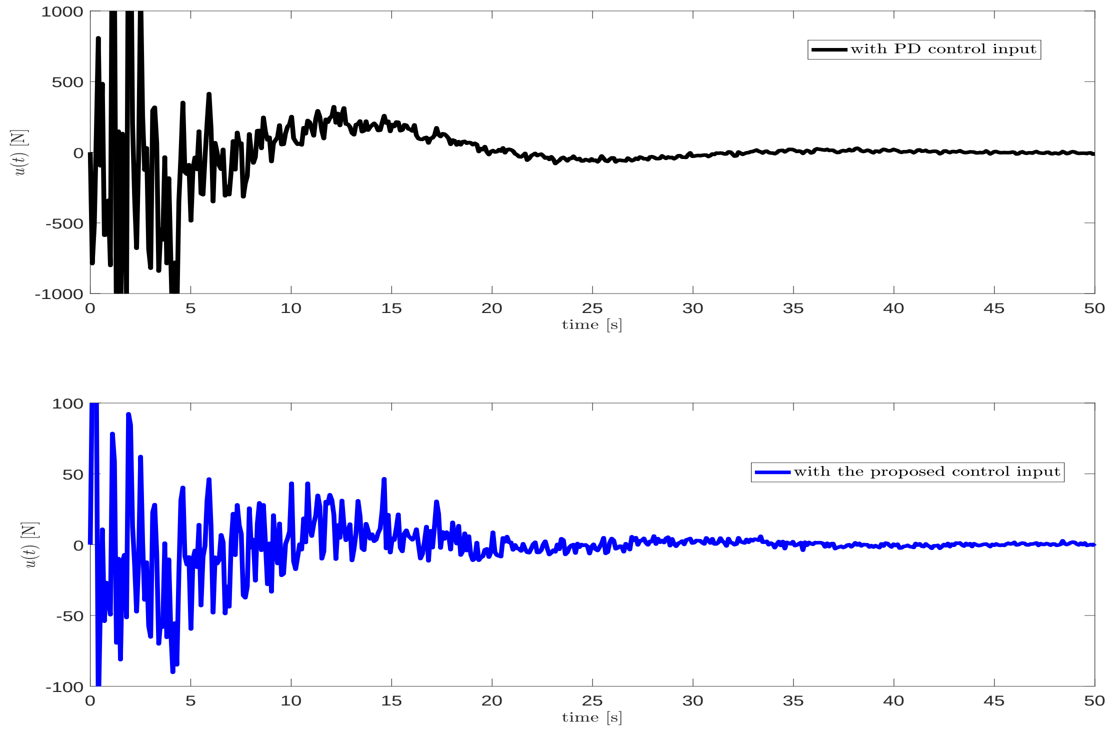

Figure 4: The diagram of control input under the two control methods.

The response of control input under the BCPD methods and BCBS methods are shown in Figure 7. Figure 7 compares the control inputs under PD control and the proposed control method. The PD control input (top) exhibits large oscillations with peaks reaching up to 1000 N , indicating high control effort and potentially inefficient actuator usage. In contrast, the proposed control (bottom) limits the peaks to about 50 N , representing a 95% reduction in control input magnitude. Additionally, the proposed control shows smoother and more stable input throughout the process, suggesting lower energy consumption and reduced actuator stress. This demonstrates that the proposed control method is significantly more efficient, requiring much less control effort while still achieving effective system stabilization.

We hope that our detailed responses and the proposed revisions adequately address the reviewers' valuable comments. We are committed to incorporating these changes to significantly enhance the manuscript's rigor, clarity, and practical relevance. We thank the editor and reviewers again for their time and constructive feedback.

2. **Comment 2:** *"Words like "superiority", "much better", "well", "is smaller" (Page 16) are very subjective and vague. How much 'better' is better? How much 'smaller' is small?"*

   Response:
   Thank you for your valuable suggestions. Regarding the terms you mentioned, such as "superiority," "much better," "well," and "is smaller," we acknowledge that they carry a certain degree of subjectivity and ambiguity. We have revised these expressions in the text to make them more objective and quantifiable.

   ......

   The responses of disturbance $d(t)$, its estimation and disturbance error $e_{d(t)}$ against a constant disturbance in Figure 5. Different from Figure 5, the responses of disturbance $d(t)$, its estimation and disturbance error $e_{d(t)}$ against time-varying disturbance in Figure 6. From Figure 5 and Figure 6, it can be observed that, for both constant and time-varying disturbances, the disturbance observer designed in this paper accurately estimates the disturbance, with the disturbance estimation error $e_{d(t)}$ converging to near zero. These results indicate that the proposed disturbance observer possesses effective disturbance estimation capability under different disturbance conditions. The response of control input under the BCPD methods and BCBS methods are shown in Figure 7. Figure 7 compares the control inputs under PD control and the proposed control method. The PD control input (top) exhibits large oscillations with peaks reaching up to 1000 N , indicating high control effort and potentially inefficient actuator usage. In contrast, the proposed control (bottom) limits the peaks to about 50 N, representing a 95% reduction in control input magnitude. Additionally, the proposed control shows smoother and more stable input throughout the process, suggesting lower energy consumption and reduced actuator stress. This demonstrates that the proposed control method is significantly more efficient, requiring much less control effort while still achieving effective system stabilization.

   We hope these modifications will help make the statements in the paper clearer and more scientific. Thank you again for your valuable feedback — we will continue working to improve the content of the article.

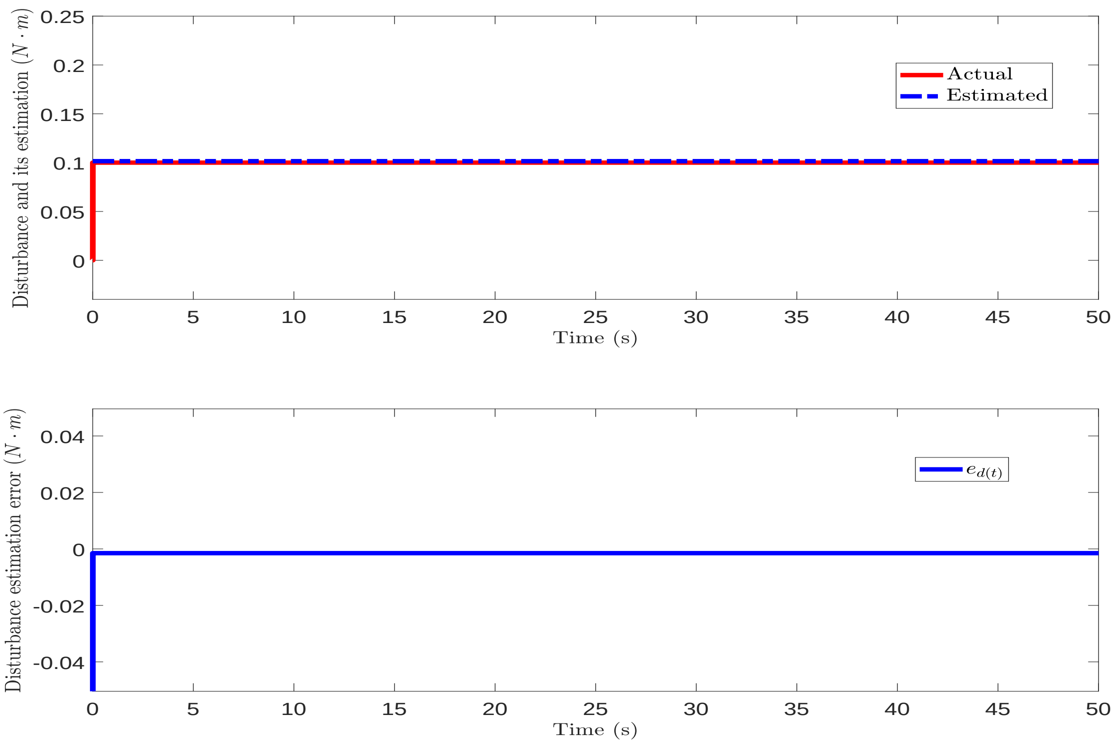

Figure 5: The responses of disturbance $d(t)$, its estimation and disturbance error against a constant disturbance.

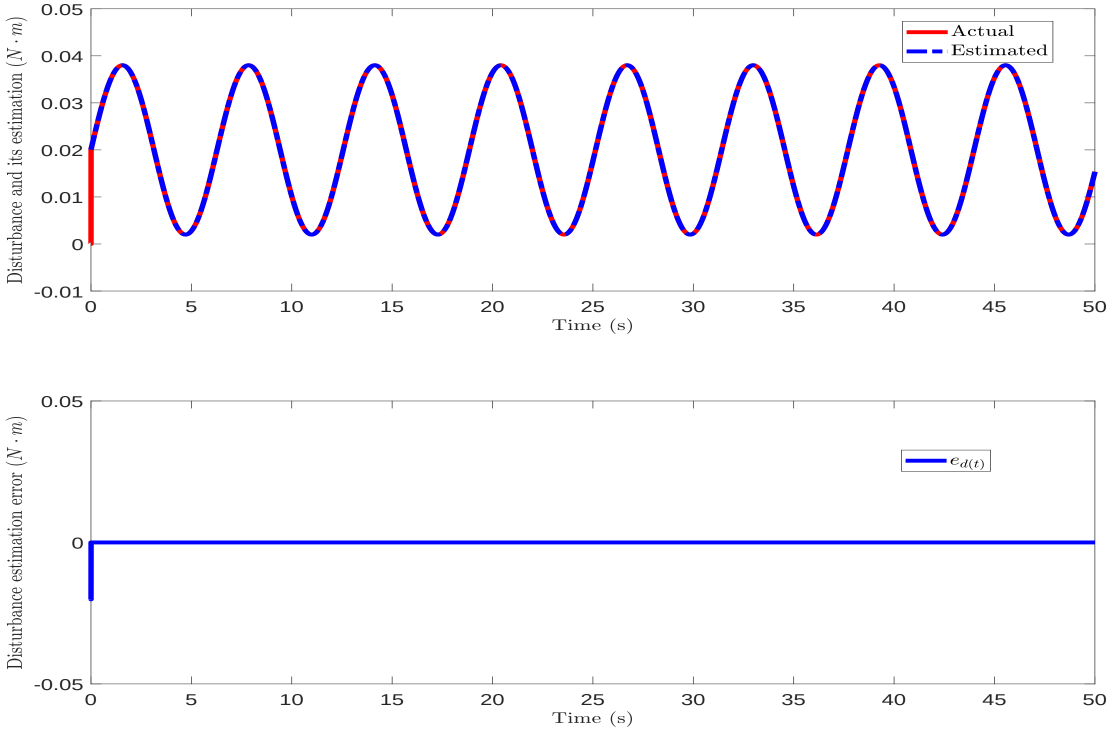

Figure 6: The responses of disturbance $d(t)$, its estimation and disturbance error against a time-varying disturbance.

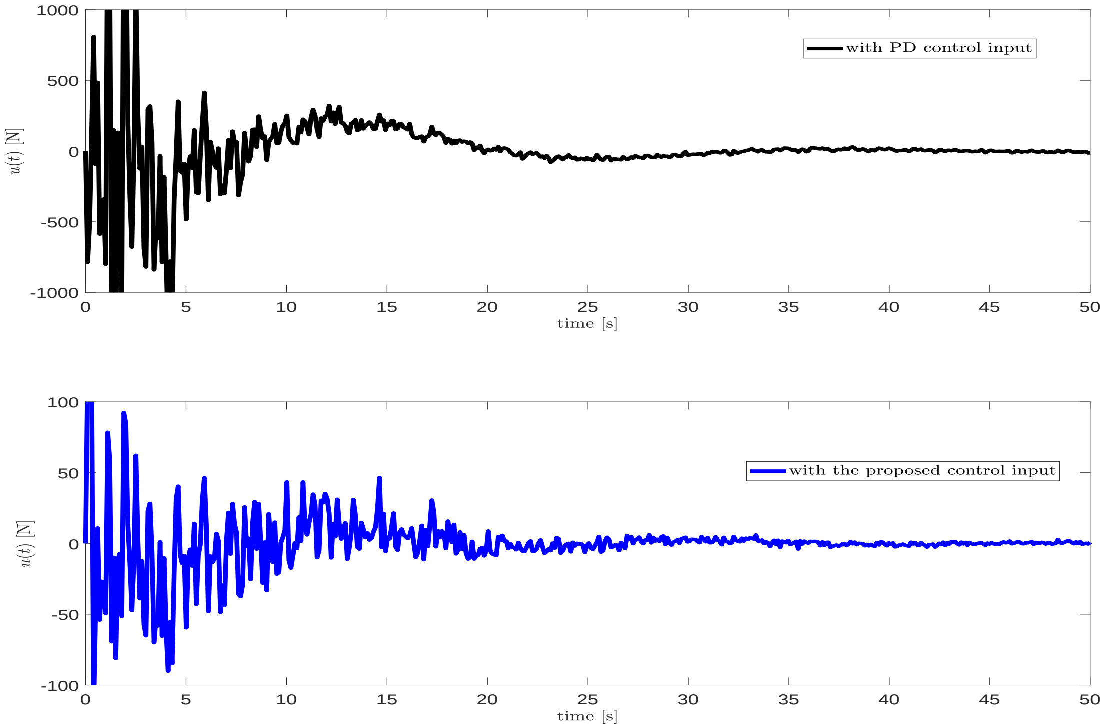

Figure 7: The diagram of control input under the two control methods.

3. **Comment 3:** *" Briefly introduce a Backstepping control wth reference to literature such as 'Adaptive backstepping integral sliding mode control of a MIMO separately excited DC Motor'. Also, mention the applications of backstepping control for example, in realising fault tolerant control schemes. See the work 'Adaptive Backstepping Based Sensor and Actuator Fault Tolerant Control of a Manipulator."*

   Response:

   Thank you for your constructive suggestion. In the revised manuscript, we have included a brief introduction to the backstepping control method, highlighting its recursive design structure for nonlinear systems and its robustness enhancement when integrated with techniques such as sliding mode control, as discussed in [Awan et al., Adaptive Backstepping Integral Sliding Mode Control of a MIMO Separately Excited DC Motor]. Additionally, we have mentioned the application of backstepping control in fault-tolerant schemes, particularly in handling actuator and sensor faults, referencing the work of Awan et al., Adaptive Backstepping Based Sensor and Actuator Fault Tolerant Control of a Manipulator. These additions have been made in the Introduction to improve the technical background and motivation for our control approach.

   [1] Afifa R, Ali S, Pervaiz M, et al. Adaptive backstepping integral sliding mode control of a mimo separately excited DC motor[J]. Robotics, 2023, 12(4): 105.

   [2] Awan Z S, Ali K, Iqbal J, et al. Adaptive backstepping based sensor and actuator fault tolerant control of a manipulator[J]. Journal of Electrical Engineering & Technology, 2019, 14(6): 2497-2504.

   ......

   Introduction

   ......

   Different from other traditional DO (see Reference [3], Reference [4] and Reference [5]), Takagi-Sugeno fuzzy method is used to describe the flexible model of flexible solar panels. The simulation also proves that this method has good stability and robustness. Awan, Zainab Shahid and Ali, Khurram et al. proposed a hybrid fault-tolerant control scheme for a 6-DOF robotic manipulator, integrating nonlinear observers, sensor redundancy, and adaptive backstepping-based actuator fault estimation with controller reconfiguration, validated by LabVIEW simulations for improved tracking under faults[2]. In Reference [6], according to the dynamic characteristics of microsatellite, a new compound controller based on sliding mode control and finite time DO is proposed in the inner loop. In [1], a robust nonlinear hybrid control for a MIMO separately excited DC motor, combining adaptive backstepping and integral sliding mode control to handle uncertainties and disturbances, with simulation results confirming improved tracking and reduced settling time compared to conventional methods. Although ODEs are simple in model description and controller design, it can not accurately describe the physical dynamic characteristics of flexible systems, which easily leads to control overflow.

   .....

   Thank you once again for your invaluable comments, which have undoubtedly helped improve the depth and quality of our manuscript.

4. **Comment 4:** *" Thoroughly analyse the computational complexity of using PDE (in contrast to ODEs) in your result. This is important to show the other side of the picture, i.e., the cons of using PDEs."*

Response:

Thank you for your valuable feedback. We appreciate your suggestion to analyze the computational complexity of using PDEs, particularly in contrast to ODEs. We agree that this is an important aspect, and we have made sure to address it in the revised manuscript to provide a more comprehensive view of the pros and cons of using PDEs.

Modification

......

Introduction

......

There are many research results on the description of system dynamic characteristics based on partial differential equations (PDEs). In Reference [7], the flexible satellite dynamics is modeled as a combination of coupled ordinary differential equations (ODEs) and PDEs using Hamilton's principle. Then, a fault-tolerant control (FTC) strategy based on adaptive integral sliding mode is proposed to address problems such as inertial uncertainty and external disturbances. In Reference [8], the use of basic PDE models and boundary control helps simplify the dynamic modeling of a small robot with flexible wings. Similarly, Reference [9] derives nonlinear coupled PDEs for the dynamics of a satellite propellant tank using Hamilton's principle, and applies a PDE-based controller to suppress slosh instability. In Reference [10], flexible solar panels are modeled as symmetric Euler-Bernoulli beams using PDEs, and a composite controller combining Nussbaum-type functions and backstepping is developed to suppress vibration under input constraints and external disturbances. Reference [11] also adopts a PDE-based infinite-dimensional modeling framework for flexible structures, similar to [7] and [10], and designs a boundary control-based FTC scheme.

While PDE-based modeling enables high-fidelity representation of distributed-parameter systems, especially in flexible structures and fluid-structure interactions, it inevitably increases the computational complexity. Compared to ODE-based lumped models, PDE models result in infinite-dimensional systems that require spatial discretization techniques (e.g., finite element, finite difference, or spectral methods) for numerical simulation and control implementation. Moreover, there are many literatures on control methods for suppressing flexible spacecraft in the presence of external disturbances, such as disturbance observer based control (DOBC) (see Reference [12], Reference [13] and Reference [14]) , active disturbance rejection control (ADRC) (Reference [15] and Reference [16]) and so on.

Thank you once again for your insightful suggestions.

5. **Comment 5:** *" What are the real-world implications of the assumptions made in this study? e.g., Assumption 1 is about the bounded nature of unknown disturbances. Is it realistic to assume in reality?"*

Response:

Thank you for your insightful comment regarding Assumption 1 and the real-world impli-

cations of the assumptions in our study. We fully appreciate the importance of considering the practical aspects of these assumptions.

Assumption 1 , which assumes that the external disturbances $d(t)$ and its derivative $\dot{d}(t)$ are bounded, is based on the understanding that real-world disturbances, though unpredictable, generally exhibit certain bounds within a specified timeframe. While it is true that disturbances in practical scenarios can vary in magnitude, assuming that they remain bounded is a common approach in control theory to ensure the stability and feasibility of the system. This assumption is particularly relevant in systems where disturbances are not entirely known but are constrained within certain limits, such as aerodynamic forces on spacecraft, sensor noise, or environmental conditions like gravitational forces or solar radiation.

In reality, it may not always be possible to exactly quantify these disturbances, but their bounded nature is often a reasonable simplification that allows for effective control and stability analysis. Moreover, by assuming that both $|d(t)| \leq \Re$ and $|\dot{d}(t)| \leq d_m$, we provide a conservative yet practical model that ensures the system can respond effectively to unforeseen forces without significant degradation in performance.

In response to your comment, we have slightly refined Assumption 1 to better reflect the bounds of disturbances in real-world systems:

......

Modification

......

Revised Assumption 1: The external disturbances $d(t)$ represent all disturbances acting on the flexible spacecraft and are assumed to be bounded within certain limits. These disturbances, including their derivatives $\dot{d}(t)$, are considered to have an upper bound in magnitude, meaning that $|d(t)| \leq \Re$ and $|\dot{d}(t)| \leq d_m$, where $\Re$ and d_m are finite values that can be determined based on system specifications and environmental factors.

We believe this revised assumption better reflects the practical limitations and conditions of real-world systems while maintaining the robustness of the model.

Thank you again for your valuable feedback. We hope that this revision clarifies the real-world applicability of our assumptions.

6. **Comment 6:** *"Include the version of MATLAB/Simulink used. Also, mention the specifications of the machines used to run the simulations in Section 4?"*

Response:

Thank you for your valuable feedback. We have updated the manuscript as per your suggestion to include the details of the MATLAB/Simulink version and the specifications of the machine used for running the simulations in Section 4.

The version of MATLAB/Simulink used for the simulations is MATLAB R2021b].

The specifications of the machine used are as follows:

- Processor: 11th Gen Intel(R) Core(TM) i7-1165G7 @ 2.80 GHz

- RAM: 16.0 GB (15.7 GB usable)

- Graphics: Intel(R) Iris(R) Xe Graphics (128 MB)

- Storage: 477 GB SSD (Samsung MZALQ512HALU-000L1)

- System Type: 64-bit operating system, based on x64 processor

In this paper, the simulation is based on MATLAB/SIMULINK, with a simulation period of 50s and a simulation step of 0.001 seconds. We have updated the manuscript to include the MATLAB/Simulink version used for the simulations and the specifications of the machine. The simulations were run on a system with an 11th Gen Intel(R) Core(TM) i7-1165G7 processor at 2.80 GHz, 16.0 GB of RAM (15.7 GB usable), Intel(R) Iris(R) Xe Graphics (128 MB), and a 477 GB SSD (Samsung MZALQ512HALU-000L1). The operating system is 64-bit based on an x64 processor.

I hope this response adequately addresses your questions. Please let me know if further clarification is needed.

7. **Comment 7:** *"How have the parameters given in Table 1 been selected?"*

   Response:

   Thank you for your comment. The parameters presented in Table 1 have been selected based on a combination of experimental data and values reported in the literature. Specifically, some of the parameters, such as the mass of the flexible spacecraft m and the density of the hose material ρ, were obtained from previously published studies, as referenced in [[17]]. Other parameters, like the Young's modulus E and elastic damping coefficient Γ_1, were chosen to match typical values used in similar flexible spacecraft models and simulations.

   We have ensured that these values are realistic and representative of typical systems in this field, and they serve as reasonable approximations for the purposes of this study.

   Thank you again for your valuable feedback.

8. **Comment 8:** *"The paper outlines on Page 3 uses Roman numbers I, II, III, while the actual section numbering writes 1, 2, 3 and so on. Please make them consistent."*

   Response:
   Thank you for your helpful observation. We apologize for the inconsistency between the Roman numerals and the Arabic numerals used in the section numbering. We have revised the manuscript to ensure that the section numbering is consistent throughout. Specifically, we have updated the outlines on Page 3 to align with the Arabic numerals used in the rest of the paper.

   ......

   Modification

   ......

   Introduction

The structure of the paper is organized as follows: In Section 1, we provide an overview of the problem description and the analysis of the flexible hose model using PDEs. Section 2 introduces a novel backstepping control strategy that utilizes boundary conditions and DO to enhance control performance. In Section 3, we present a comparative study between the proposed control method and a traditional PD control approach through simulation examples, demonstrating the effectiveness and advantages of our proposed strategy. Finally, Section 4 offers concluding remarks and discusses potential directions for future work in this field.

We appreciate your attention to detail and hope this adjustment improves the clarity of the manuscript.

9. **Comment 9:** *"The discussion on disturbance observer at the start of Section 3.1 could benefit from the 'Disturbance-observer-based robust control of a Serial-link robotic manipulator using SMC and PBC techniques."*

   Response:
   Thank you for your valuable suggestion. We appreciate your recommendation to incorporate the literature "Disturbance-observer-based robust control of a Serial-link robotic manipulator using SMC and PBC techniques" into the discussion on disturbance observers in Section 3.1.

   We have reviewed the suggested paper and have included relevant references and insights from it to enrich the discussion on the design of the disturbance observer (DO). The cited work has been referenced in the revised version, and we have expanded the section to highlight the connection between our approach and previous methods in disturbance observer-based robust control, particularly in the context of using Sliding Mode Control (SMC) and Passivity-Based Control (PBC) techniques.

   [18] Ajwad S A, Iqbal J, Khan A A, et al. Disturbance-observer-based robust control of a serial-link robotic manipulator using SMC and PBC techniques[J]. Studies in Informatics and Control, 2015, 24(4): 401-408.

   ......

   Modification

   ......

   3.1 Design of disturbance observer

   In this section, a DO is designed to estimate the disturbances $d(t)$ in the flexible spacecraft system. The estimated disturbance is denoted as $\hat{d}(t)$, and the derivative of the external disturbance estimation is given by [18]

   We believe this addition strengthens the paper and provides a more comprehensive understanding of the disturbance observer design.

10. **Comment 10:** *" Please thoroughly proofread the whole paper for typos, spelling mistakes and other linguistic improvements. A FEW examples are given below:*

Response:
Thank you for your valuable feedback and suggestions. I sincerely appreciate your effort in reviewing the manuscript.

I have carefully proofread the entire paper, addressing all typographical errors, spelling mistakes, and linguistic improvements as suggested. The necessary corrections have been made to enhance the clarity and quality of the paper.

Once again, I appreciate your constructive comments, which have certainly helped improve the manuscript. If you have any further suggestions or concerns, please do not hesitate to let me know.

11. **Comment 11:** *" "Ploytechnic" in the affiliation of the first author "*

    Response:
    Thank you for pointing out the typo in the affiliation of the first author. I apologize for the oversight. The term "Ploytechnic" has been corrected to "Polytechnic" in the manuscript.

    I appreciate your attention to detail, and the necessary correction has been made.

12. **Comment 12:** *"... which can accurately describes the ..." (describe)*

    Response:
    Thank you for your insightful comment. I have carefully reviewed the sentence and noticed the grammatical issue. The sentence has been revised to ensure proper verb usage:

    "First, a partial differential equation (PDE) with boundary initial conditions is derived using Hamilton's principle, accurately describing the dynamic characteristics of the flexible structure."

    I appreciate your attention to detail, and the necessary correction has been made.

The authors wish to extend their heartfelt gratitude to the referee for their valuable and insightful feedback. The constructive yet critical comments provided by the referee have been instrumental in refining both the quality and the clarity of this work. Their thoughtful suggestions and detailed efforts have significantly contributed to enhancing the overall presentation and rigor of the paper, for which we are deeply appreciative.

References

[1] R. Afifa, S. Ali, M. Pervaiz, and J. Iqbal, "Adaptive backstepping integral sliding mode control of a mimo separately excited dc motor," *Robotics*, vol. 12, no. 4, p. 105, 2023.

[2] Z. S. Awan, K. Ali, J. Iqbal, and A. Mehmood, "Adaptive backstepping based sensor and actuator fault tolerant control of a manipulator," *Journal of Electrical Engineering & Technology*, vol. 14, no. 6, pp. 2497–2504, 2019.

[3] S. Cao, Y. Yi, and L. Guo, "Anti-disturbance fault diagnosis for non-gaussian stochastic distribution systems with multiple disturbances," *Neurocomputing*, vol. 136, pp. 315–320, 2014.

[4] F. Cao and J. Liu, “Adaptive neural network control of an arm-string system with actuator fault based on a pde model,” *Journal of Vibration and Control*, vol. 25, no. 1, pp. 172–181, 2019.

[5] Y. Qu, X. Zhong, F. Zhang, X. Tong, L. Fan, and L. Dai, “Robust disturbance observer-based fast maneuver method for attitude control of optical remote sensing satellites,” *Acta Astronautica*, vol. 201, pp. 83–93, 2022.

[6] J. Qiao, D. Zhang, Y. Zhu, and P. Zhang, “Disturbance observer-based finite-time attitude maneuver control for micro satellite under actuator deviation fault,” *Aerospace science and technology*, vol. 82, pp. 262–271, 2018.

[7] L. Ashayeri, A. Doustmohammadi, and F. F. Saberi, “Fault-tolerant control of flexible satellite with infinite-dimensional model,” *Advances in Space Research*, vol. 68, no. 7, pp. 3080–3092, 2021.

[8] A. A. Paranjape, J. Guan, S.-J. Chung, and M. Krstic, “Pde boundary control for flexible articulated wings on a robotic aircraft,” *IEEE Transactions on Robotics*, vol. 29, no. 3, pp. 625–640, 2013.

[9] M. Jokar, H. Salarieh, and H. N. Pishkenari, “Satellite attitude pde-based controller design considering fluid fuel sloshing in planar maneuver,” *Aerospace Science and Technology*, vol. 122, p. 107366, 2022.

[10] N. Ji and J. Liu, “Vibration control for a flexible satellite with input constraint based on nussbaum function via backstepping method,” *Aerospace Science and Technology*, vol. 77, pp. 563–572, 2018.

[11] M. M. Ataei, H. Salarieh, H. Nejat Pishkenari, and H. Jalili, “Boundary control design based on partial differential equation observer for vibration suppression and attitude control of flexible satellites with multi-section solar panels,” *Journal of Vibration and Control*, p. 1077546321990158, 2021.

[12] S. Cao and B. Hang, “Adaptive fault tolerant attitude control of flexible satellites based on takagi-sugeno fuzzy disturbance modeling,” *Transactions of the Institute of Measurement and Control*, vol. 42, no. 9, pp. 1712–1723, 2020.

[13] A. Noshadi, J. Shi, W. S. Lee, P. Shi, and A. Kalam, “Robust control of an active magnetic bearing system using h_∞ and disturbance observer-based control,” *Journal of Vibration and Control*, vol. 23, no. 11, pp. 1857–1870, 2017.

[14] B. Xu, D. Wang, Y. Zhang, and Z. Shi, “Dob-based neural control of flexible hypersonic flight vehicle considering wind effects,” *IEEE Transactions on Industrial Electronics*, vol. 64, no. 11, pp. 8676–8685, 2017.

[15] H. Fang, X. Yuan, and P. Liu, “Active–disturbance–rejection–control and fractional–order–proportional–integral–derivative hybrid control for hydroturbine speed governor system,” *Measurement and Control*, vol. 51, no. 5-6, pp. 192–201, 2018.

[16] Y. Zhang, Z. Chen, and M. Sun, “Trajectory tracking control for a quadrotor unmanned aerial vehicle based on dynamic surface active disturbance rejection control,” *Transactions of the Institute of Measurement and Control*, vol. 42, no. 12, pp. 2198–2205, 2020.

[17] W. He, X. He, M. Zou, and H. Li, "Pde model-based boundary control design for a flexible robotic manipulator with input backlash," *IEEE Transactions on Control Systems Technology*, vol. 27, no. 2, pp. 790–797, 2018.

[18] S. A. Ajwad, J. Iqbal, A. A. Khan, and A. Mehmood, "Disturbance-observer-based robust control of a serial-link robotic manipulator using smc and pbc techniques," *Studies in Informatics and Control*, vol. 24, no. 4, pp. 401–408, 2015.

[19] S. Jia and J. Shan, "Finite-time trajectory tracking control of space manipulator under actuator saturation," *IEEE Transactions on Industrial Electronics*, vol. 67, no. 3, pp. 2086–2096, 2019.

[20] T. Chen and J. Shan, "Distributed control of multiple flexible manipulators with unknown disturbances and dead-zone input," *IEEE Transactions on Industrial Electronics*, vol. 67, no. 11, pp. 9937–9947, 2019.

[21] A. Bounemeur, M. chemachema, A. Zahaf, and S. Bououden, "Adaptive fuzzy fault-tolerant control using nussbaum gain for a class of siso nonlinear systems with unknown directions," in *Proceedings of the 4th International Conference on Electrical Engineering and Control Applications: ICEECA 2019, 17–19 December 2019, Constantine, Algeria.* Springer, 2021, pp. 493–510.

[22] A. Bounemeur and M. Chemachema, "Adaptive fuzzy fault-tolerant control using nussbaum-type function with state-dependent actuator failures," *Neural Computing and Applications*, vol. 33, pp. 191–208, 2021.

[23] O. Bey and M. Chemachema, "Finite-time event-triggered output-feedback adaptive decentralized echo-state network fault-tolerant control for interconnected pure-feedback nonlinear systems with input saturation and external disturbances: A fuzzy control-error approach," *Information Sciences*, vol. 669, p. 120557, 2024.

[24] A. Bounemeur and M. Chemachema, "Finite-time output-feedback fault tolerant adaptive fuzzy control framework for a class of mimo saturated nonlinear systems," *International Journal of Systems Science*, pp. 1–20, 2024.

[25] Y.-C. Liu, S. Laghrouche, A. N'Diaye, and M. Cirrincione, "Hermite neural network-based second-order sliding-mode control of synchronous reluctance motor drive systems," *Journal of the Franklin Institute*, vol. 358, no. 1, pp. 400–427, 2021.

[26] F.-J. Lin, M.-S. Huang, Y.-C. Chien, and S.-G. Chen, "Intelligent backstepping control of permanent magnet-assisted synchronous reluctance motor position servo drive with recurrent wavelet fuzzy neural network," *Energies*, vol. 16, no. 14, p. 5389, 2023.

Response to Reviewer No. 2:

Ms. No. PONE-D-25-18264

"Active control of flexible spacecraft in orbit based on partial differential equations"

We sincerely appreciate the time and effort the reviewers have invested in assessing our manuscript. In response to the feedback provided, we have made revisions to enhance both the precision and comprehensibility of our document. To facilitate the reviewers' assessment of these changes, we have marked the sections modified in response to **Reviewer 2's comments** in **red**. We are grateful for the valuable insights that have significantly improved our manuscript.

1. **Comment 1:** *"Please add a figure to clearly illustrate the specific spacecraft system or flexible structure studied."*

   Response:
   Thank you for your valuable suggestion. We understand the importance of visual clarity in illustrating the specific spacecraft system or flexible structure studied. In response, we have added a figure to the manuscript that clearly illustrates the system and its key components. The updated figure has been incorporated into the revised version of the manuscript, and we believe it will help to better communicate the concepts and details of the study.

   Modification

   ......

   The flexible spacecraft system is mainly composed of tanker, receiver and flexible spacecraft, as shown in Figure 8 . $X_g - Y_g$ represents an inertial reference frame. $X - Y$ represents the local coordinate system and moves horizontally. $x - y$ is the body fixed coordinate system , which is attached to the connection point between the flexible spacecraft and the receiver aircraft. In this paper, only the horizontal direction of flexible spacecraft is discussed. The control input $u(t)$ represents the top boundary actuator of the flexible spacecraft. $d(t)$ represents the external disturbances to the flexible hose during flexible spacecraft. m is the mass of the actuator.

   Thank you again for your valuable feedback.

2. **Comment 2:** *"The literature review should be more comprehensive. For example, similar observers for PDEs have appeared in Prof. Wenhao's work, and Prof. Jinjun Shan has done extensive adaptive control research on flexible spacecraft. Please summarize these and other relevant works in more depth."*

   Response:

   Thank you very much for your valuable feedback. We appreciate your suggestion to expand the literature review. Based on your recommendation, we have thoroughly reviewed Prof.

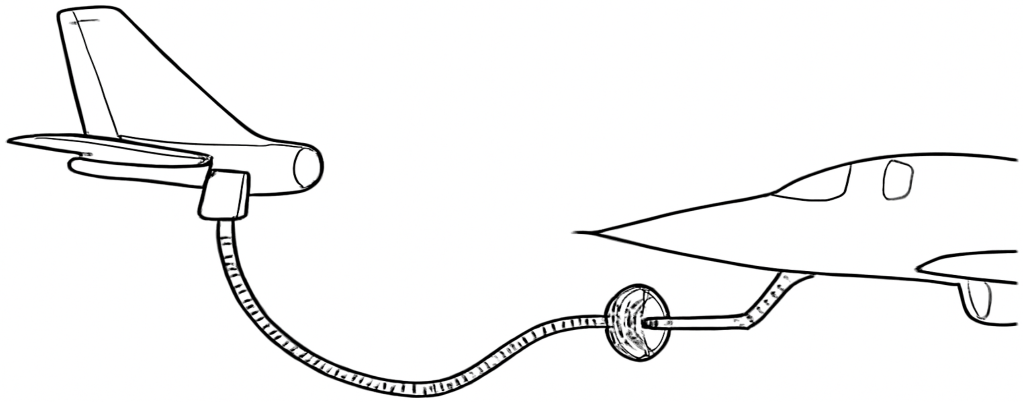

Figure 8: The diagram of flexible hose.

Wenhao's work on observers for PDEs and Prof. Jinjun Shan's extensive research on adaptive control for flexible spacecraft. We have now included a more comprehensive summary of these and other relevant studies, highlighting their contributions and how they relate to our research. We believe these additions provide a clearer context for our work and better connect it to existing literature.

......

Modification

......

Introduction

In Reference [19], a finite-time trajectory tracking controller is presented for a space manipulator, addressing model uncertainty, external disturbances, and actuator saturation. A radial basis function neural network (NN) is used to estimate model uncertainties, while an auxiliary system compensates for actuator saturation, and a NN-based adaptive terminal sliding mode controller ensures stable trajectory tracking, as confirmed by Lyapunov stability analysis and numerical simulations.

......

In Reference [20], this study presents two distributed control strategies for a team of flexible spacecraft to track the attitude of a virtual leader using a PDE observer. The first strategy uses direct state measurements from the hub and free tip of the beam, while the second incorporates a PDE observer to estimate the beam's states, with both approaches ensuring asymptotic attitude tracking without residual vibration.

......

Thank you again for your helpful input, and we hope the revised literature review addresses your concerns.

3. **Comment 3:** *"PDE modeling itself is not a novel contribution; rather, the control challenges arising from PDEs are worth deeper discussion."*

   Response:
   Thank you for your insightful feedback. We agree that while PDE modeling is a well-established approach, the primary contribution of our work lies in addressing the control challenges that arise from the PDEs governing the dynamics of flexible spacecraft under external disturbances and input constraints. In response to your comment, we have expanded the discussion on the specific control challenges posed by the PDEs, particularly focusing on the complexities involved in designing a stable and efficient controller in the presence of such dynamics. We believe this additional discussion better highlights the novelty and significance of our work in the context of flexible spacecraft control.

   Thank you again for your valuable suggestion, and we hope that the revised manuscript more clearly emphasizes the control challenges and solutions presented in our study.

4. **Comment 4:** *"The solution method section needs more details. MATLAB is just a platform—please further explain the actual numerical or computational techniques used."*

   Response:
   Thank you for your constructive feedback. We appreciate your suggestion to provide more details in the solution method section. In response, we have expanded the explanation of the numerical and computational techniques used in the study. Specifically, we now provide a detailed description of the algorithms and methods implemented in MATLAB, including the numerical integration approach, the handling of PDEs, and the stability analysis. Additionally, we have clarified the role of the computer's performance specifications in ensuring the efficiency and accuracy of the simulations.

   Modification

   ......

   Simulation Examples

   ......

   In this paper, the simulation is based on MATLAB/SIMULINK, with a simulation period of 50s and a simulation step of 0.001 seconds. We have updated the manuscript to include the MATLAB/Simulink version used for the simulations and the specifications of the machine. The simulations were run on a system with an 11th Gen Intel(R) Core(TM) i7-1165G7 processor at 2.80 GHz, 16.0 GB of RAM (15.7 GB usable), Intel(R) Iris(R) Xe Graphics (128 MB), and a 477 GB SSD (Samsung MZALQ512HALU-000L1). The operating system is 64-bit based on an x64 processor.

   We believe that these additions will make the computational techniques more transparent and help readers better understand the implementation of our solution.

5. **Comment 5:** *" The assumption on the disturbance type should be discussed."*

   Response:

Thank you for your valuable feedback. We appreciate your suggestion to further discuss the disturbance type assumed in our study. In response, we have expanded the discussion on the nature of the external disturbances $d(t)$. Specifically, we clarify that the disturbances are considered to be bounded and include both deterministic and possibly unknown components. These disturbances, as outlined in Assumption 1, are assumed to have finite upper bounds on their magnitudes and derivatives, which is a common assumption for modeling external disturbances in practical systems. Additionally, we have discussed the implications of this assumption in the context of flexible spacecraft, emphasizing that it is based on typical environmental factors such as solar radiation, gravitational forces, and aerodynamic effects in space environments.

......

Modification

......

Assumption 1

Assumption 1: The external disturbances $d(t)$ represent all disturbances acting on the flexible spacecraft and are assumed to be bounded within certain limits. These disturbances, including their derivatives $\dot{d}(t)$, are considered to have an upper bound in magnitude, meaning that $|d(t)| \leq \Re$ and $|\dot{d}(t)| \leq d_m$, where $\Re$ and d_m are finite values that can be determined based on system specifications and environmental factors.

We hope this added clarification helps to better contextualize the assumptions made regarding disturbances in our study.

Thank you again for your insightful suggestion.

6. **Comment 6:** *" PLOS authors have the option to publish the peer review history of their article (what does this mean?). If published, this will include your full peer review and any attached files. If you choose "no", your identity will remain anonymous but your review may still be made public.'*

   Response: Thank you for your clarification regarding the peer review history. We understand that PLOS authors have the option to publish the peer review history of their article, which includes the full peer review process and any attached files. After carefully considering this, we would prefer to keep our identity anonymous, and thus, we choose the "no" option. We understand that while our identity will remain anonymous, the review process may still be made public.

   Thank you for providing this option and for your understanding.

   The authors sincerely appreciate the reviewer's detailed and insightful feedback, which has significantly contributed to improving both the quality and clarity of our manuscript. We are truly grateful for the time and effort invested in offering constructive criticism and valuable suggestions, helping us enhance both the substance and presentation of our work.

References

[1] R. Afifa, S. Ali, M. Pervaiz, and J. Iqbal, "Adaptive backstepping integral sliding mode control of a mimo separately excited dc motor," *Robotics*, vol. 12, no. 4, p. 105, 2023.

[2] Z. S. Awan, K. Ali, J. Iqbal, and A. Mehmood, "Adaptive backstepping based sensor and actuator fault tolerant control of a manipulator," *Journal of Electrical Engineering & Technology*, vol. 14, no. 6, pp. 2497–2504, 2019.

[3] S. Cao, Y. Yi, and L. Guo, "Anti-disturbance fault diagnosis for non-gaussian stochastic distribution systems with multiple disturbances," *Neurocomputing*, vol. 136, pp. 315–320, 2014.

[4] F. Cao and J. Liu, "Adaptive neural network control of an arm-string system with actuator fault based on a pde model," *Journal of Vibration and Control*, vol. 25, no. 1, pp. 172–181, 2019.

[5] Y. Qu, X. Zhong, F. Zhang, X. Tong, L. Fan, and L. Dai, "Robust disturbance observer-based fast maneuver method for attitude control of optical remote sensing satellites," *Acta Astronautica*, vol. 201, pp. 83–93, 2022.

[6] J. Qiao, D. Zhang, Y. Zhu, and P. Zhang, "Disturbance observer-based finite-time attitude maneuver control for micro satellite under actuator deviation fault," *Aerospace science and technology*, vol. 82, pp. 262–271, 2018.

[7] L. Ashayeri, A. Doustmohammadi, and F. F. Saberi, "Fault-tolerant control of flexible satellite with infinite-dimensional model," *Advances in Space Research*, vol. 68, no. 7, pp. 3080–3092, 2021.

[8] A. A. Paranjape, J. Guan, S.-J. Chung, and M. Krstic, "Pde boundary control for flexible articulated wings on a robotic aircraft," *IEEE Transactions on Robotics*, vol. 29, no. 3, pp. 625–640, 2013.

[9] M. Jokar, H. Salarieh, and H. N. Pishkenari, "Satellite attitude pde-based controller design considering fluid fuel sloshing in planar maneuver," *Aerospace Science and Technology*, vol. 122, p. 107366, 2022.

[10] N. Ji and J. Liu, "Vibration control for a flexible satellite with input constraint based on nussbaum function via backstepping method," *Aerospace Science and Technology*, vol. 77, pp. 563–572, 2018.

[11] M. M. Ataei, H. Salarieh, H. Nejat Pishkenari, and H. Jalili, "Boundary control design based on partial differential equation observer for vibration suppression and attitude control of flexible satellites with multi-section solar panels," *Journal of Vibration and Control*, p. 1077546321990158, 2021.

[12] S. Cao and B. Hang, "Adaptive fault tolerant attitude control of flexible satellites based on takagi-sugeno fuzzy disturbance modeling," *Transactions of the Institute of Measurement and Control*, vol. 42, no. 9, pp. 1712–1723, 2020.

[13] A. Noshadi, J. Shi, W. S. Lee, P. Shi, and A. Kalam, "Robust control of an active magnetic bearing system using h_∞ and disturbance observer-based control," *Journal of Vibration and Control*, vol. 23, no. 11, pp. 1857–1870, 2017.

[14] B. Xu, D. Wang, Y. Zhang, and Z. Shi, "Dob-based neural control of flexible hypersonic flight vehicle considering wind effects," *IEEE Transactions on Industrial Electronics*, vol. 64, no. 11, pp. 8676–8685, 2017.

[15] H. Fang, X. Yuan, and P. Liu, "Active–disturbance–rejection–control and fractional–order–proportional–integral–derivative hybrid control for hydroturbine speed governor system," *Measurement and Control*, vol. 51, no. 5-6, pp. 192–201, 2018.

[16] Y. Zhang, Z. Chen, and M. Sun, "Trajectory tracking control for a quadrotor unmanned aerial vehicle based on dynamic surface active disturbance rejection control," *Transactions of the Institute of Measurement and Control*, vol. 42, no. 12, pp. 2198–2205, 2020.

[17] W. He, X. He, M. Zou, and H. Li, "Pde model-based boundary control design for a flexible robotic manipulator with input backlash," *IEEE Transactions on Control Systems Technology*, vol. 27, no. 2, pp. 790–797, 2018.

[18] S. A. Ajwad, J. Iqbal, A. A. Khan, and A. Mehmood, "Disturbance-observer-based robust control of a serial-link robotic manipulator using smc and pbc techniques," *Studies in Informatics and Control*, vol. 24, no. 4, pp. 401–408, 2015.

[19] S. Jia and J. Shan, "Finite-time trajectory tracking control of space manipulator under actuator saturation," *IEEE Transactions on Industrial Electronics*, vol. 67, no. 3, pp. 2086–2096, 2019.

[20] T. Chen and J. Shan, "Distributed control of multiple flexible manipulators with unknown disturbances and dead-zone input," *IEEE Transactions on Industrial Electronics*, vol. 67, no. 11, pp. 9937–9947, 2019.

[21] A. Bounemeur, M. chemachema, A. Zahaf, and S. Bououden, "Adaptive fuzzy fault-tolerant control using nussbaum gain for a class of siso nonlinear systems with unknown directions," in *Proceedings of the 4th International Conference on Electrical Engineering and Control Applications: ICEECA 2019, 17–19 December 2019, Constantine, Algeria.* Springer, 2021, pp. 493–510.

[22] A. Bounemeur and M. Chemachema, "Adaptive fuzzy fault-tolerant control using nussbaum-type function with state-dependent actuator failures," *Neural Computing and Applications*, vol. 33, pp. 191–208, 2021.

[23] O. Bey and M. Chemachema, "Finite-time event-triggered output-feedback adaptive decentralized echo-state network fault-tolerant control for interconnected pure-feedback nonlinear systems with input saturation and external disturbances: A fuzzy control-error approach," *Information Sciences*, vol. 669, p. 120557, 2024.

[24] A. Bounemeur and M. Chemachema, "Finite-time output-feedback fault tolerant adaptive fuzzy control framework for a class of mimo saturated nonlinear systems," *International Journal of Systems Science*, pp. 1–20, 2024.

[25] Y.-C. Liu, S. Laghrouche, A. N'Diaye, and M. Cirrincione, "Hermite neural network-based second-order sliding-mode control of synchronous reluctance motor drive systems," *Journal of the Franklin Institute*, vol. 358, no. 1, pp. 400–427, 2021.

[26] F.-J. Lin, M.-S. Huang, Y.-C. Chien, and S.-G. Chen, "Intelligent backstepping control of permanent magnet-assisted synchronous reluctance motor position servo drive with recurrent wavelet fuzzy neural network," *Energies*, vol. 16, no. 14, p. 5389, 2023.
